# Achieving Automated Early Detection: Update on LANGaware's Language and Speech Biomarkers in Neurocognitive and Affective Disorders

**DOI:** 10.1002/alz70856_104966

**Published:** 2026-01-08

**Authors:** Vassiliki Rentoumi, Evangelos Vassiliou, Admir Demiraj, Manolis Papageorgiou, Dimitra Sali, Magda Tsolaki, John D Papatriantafyllou, George Paliouras

**Affiliations:** ^1^ LANGaware Inc, Menlo Park, CA, USA; ^2^ Department of Financial and Management Engineering, School of Engineering, University of the Aegean, Chios, Greece; ^3^ LANGaware Inc, Menlo Park, USA, Menlo Park, CA, USA; ^4^ Aiginiteio University Hospital, Athens, Greece; ^5^ Greek Association of Alzheimer Disease and Related Disorders, Thessaloniki, Greece; ^6^ IASIS ‐ Day Care Center for Mental Disorders of Third Age, Athens, Greece; ^7^ NCSR 'Demokritos', Athens, Greece

## Abstract

**Background:**

Advances in automated language and speech analysis using machine learning have validated digital biomarkers for non‐invasive detection of subtle cognitive changes. While distinguishing Alzheimer's Disease (AD) from Normal Controls (NC) is straightforward, classifying Mild Cognitive Impairment (MCI) remains challenging, due to its potential progression to AD or its association with other factors such as affective disorders, requiring detailed expert evaluation. Expanding upon prior research, this study assesses LANGaware's biomarker capabilities on recent data for (a) binary classification of AD versus NC, (b) multi‐class classification into AD, NC, and MCI, and (c) binary classification of Depression (D) versus NC.

**Method:**

Diagnoses have been collected for the above cases, provided by medical experts following standardized protocols. Participants were engaged in simple elicitation tasks, such as describing a picture or narrating an event. Digital biomarkers that reflect linguistic, speech, and acoustic features were extracted from recorded audio and corresponding transcripts. These biomarkers were then used as input for classification tasks, employing a tailored neural network and an XGBoost model to perform binary and multi‐class (three‐class) classifications. The methodology was designed to be easily adaptable to multiple languages.

**Result:**

For cognitive classification, 15827 elicitation tasks (5173 AD, 7660 MCI, 2994 NC) from English‐ and Greek‐speaking participants were analyzed using nested cross‐validation. The binary classifier (AD vs. Healthy) achieved an average F1‐macro score of 89.01%, while the three‐class one (AD vs. MCI vs. NC) attained a score of 73.0%. For depression classification, 4421 elicitation tasks (1481 D, 2940 NC) from English‐speaking participants were evaluated, achieving an average F1‐macro score of 70.38%.

**Conclusion:**

The results obtained confirm the strong discriminative capability of the proposed biomarkers for the early detection of cognitive decline. The findings support the applicability of automated assessment, facilitating early diagnosis and timely intervention. Additionally, the depression classification experiment further complements the cognitive analysis, given the established link between depression and MCI. This study is crucial for ensuring the quality and reliability of the LANGaware product as it is increasingly adopted by diagnostic centers and hospitals. We express our gratitude to all organizations that contributed valuable data to this work.

